# Case Report: Bilateral optic disc edema with papillophlebitis-like features following combined cisplatin and capecitabine chemotherapy

**DOI:** 10.3389/fopht.2026.1961914

**Published:** 2026-09-28

**Authors:** Shanshan Cao, Lu Wang, Wei Chen, Yong Liu

**Affiliations:** Department of Ophthalmology, Air Force Medical Center of PLA, Beijing, China

**Keywords:** capecitabine, case report, chemotherapy-associated ocular toxicity, cisplatin, optic disc edema, papillophlebitis-like features

## Abstract

**Background:**

Ocular adverse events associated with cisplatin and capecitabine are uncommon, and bilateral optic disc edema with papillophlebitis-like features has rarely been reported. We describe a case of bilateral optic disc edema with papillophlebitis-like features following combined cisplatin and capecitabine chemotherapy in a patient with squamous cell carcinoma of the anal canal. We discuss the differential diagnosis, possible mechanisms, and clinical course in the context of the existing literature.

**Case presentation:**

A 50-year-old woman with squamous cell carcinoma of the anal canal received pelvic radiotherapy (28 fractions) combined with four cycles of cisplatin and oral capecitabine. Sixteen days after the final cycle of chemotherapy, she developed painless bilateral blurred vision. Fundus examination revealed bilateral optic disc edema and hyperemia, peripapillary flame-shaped hemorrhages, and dilated retinal veins. Fundus fluorescein angiography demonstrated leakage from the optic disc vasculature, while optical coherence tomography(OCT) showed bilateral thickening of the peripapillary retinal nerve fiber layer. Comprehensive systemic work-up, including complete blood count, inflammatory markers, tumor markers, a paraneoplastic antibody panel, contrast-enhanced brain magnetic resonance imaging, and multidisciplinary consultation, revealed no alternative systemic, neurologic, or paraneoplastic explanation. Intracranial hypertension, optic neuritis, and non-arteritic anterior ischemic optic neuropathy (NAION) were considered less likely based on the overall clinical findings. No systemic glucocorticoids were administered, and the patient was managed with close observation. At the two-month follow-up, best-corrected visual acuity had improved to 1.2 in both eyes, with complete resolution of the optic disc edema on fundus examination and OCT.

**Discussion:**

The temporal association with chemotherapy, bilateral presentation, papillophlebitis-like fundus findings, and spontaneous resolution after completion of treatment suggest a possible chemotherapy-associated toxic or vascular reaction. Cisplatin-related endothelial toxicity and capecitabine-associated vasospasm may be potential mechanisms. However, a direct causal relationship cannot be established from a single case.

## Introduction

Optic disc edema can arise from a broad range of ocular and systemic conditions and may occasionally be accompanied by retinal venous abnormalities. The combination of optic disc edema and hyperemia, peripapillary hemorrhages, and retinal venous dilatation or tortuosity may resemble papillophlebitis and can be descriptively termed a papillophlebitis-like reaction. The mechanisms underlying this clinical presentation remain incompletely understood, although inflammatory changes, increased vascular permeability, and disturbances in retinal venous circulation have been proposed (1). Reported associations include infectious, inflammatory, autoimmune, and drug-related conditions (2). However, bilateral optic disc edema with papillophlebitis-like features following chemotherapy has rarely been described.

Among platinum-based chemotherapeutic agents, cisplatin has been associated with various ocular adverse events, which can manifest as optic neuropathy, retinal damage, and optic disc edema (3). A disproportionality analysis of the US Food and Drug Administration Adverse Event Reporting System identified 69 ocular safety signals related to platinum agents, including reporting signals for optic disc hyperemia with oxaliplatin and cortical blindness with cisplatin. The same study also reviewed 32 published cases of suspected platinum-associated ocular toxicity (4). Cisplatin-induced toxic optic neuropathy has also been reported in a patient with Hodgkin's lymphoma (5). Bilateral optic disc edema has also been reported following platinum-based chemotherapy, with fundus fluorescein angiography showing diffuse optic disc leakage in the late phase (6). In 1997, bilateral optic neuropathy characterized by central scotomas and visual loss was reported following high-dose cisplatin and carboplatin chemotherapy, with gradual visual recovery over one year (7). Collectively, these reports indicate that platinum-associated ocular toxicity has been recognized for several decades, although it remains uncommon and clinically heterogeneous.

Capecitabine, an oral prodrug of 5-fluorouracil (5-FU), has been associated primarily with ocular surface irritation, corneal abnormalities, and transient visual symptoms (8). However, optic nerve or optic disc involvement has rarely been reported (9). Severe corneal toxicity has also been described during capecitabine treatment (10), and a case of severe visual loss temporally associated with capecitabine was reported in 2023 (11). Its active metabolite, 5-FU, has rarely been associated with optic neuropathy, for which vasospastic and ischemic mechanisms have been proposed. Bilateral optic disc edema and optic neuropathy have also been reported following FOLFOX therapy, which combines 5-FU, leucovorin, and oxaliplatin (12). In addition, optic neuritis has rarely been reported in association with 5-FU (13). These reports support biological plausibility, but do not establish that either agent caused the phenotype observed in this patient.

Recent clinical evidence also suggests dose-related retinal structural changes after platinum exposure. In a cohort of 36 pediatric oncology patients, higher cumulative cisplatin exposure was associated with reduced central foveal retinal thickness and thinning of the nasal-superior retinal nerve fiber layer (RNFL) (14). However, evidence specific to combined cisplatin and capecitabine therapy and bilateral papillophlebitis-like features remains limited. We therefore report this case as chemotherapy-associated bilateral optic disc edema and discuss the relevant differential diagnoses, possible mechanisms, and clinical implications.

## Case presentation

A 50-year-old woman developed bilateral blurred vision 16 days after completing four 28-day cycles of cisplatin and capecitabine chemotherapy delivered concurrently with pelvic radiotherapy for anal canal squamous cell carcinoma (cT4N1M0, stage IIIC). Pelvic radiotherapy was delivered as dose-painted intensity-modulated radiotherapy with simultaneous integrated boost, prescribed as 60 Gy in 25 fractions to the involved pelvic lymph nodes (PGTVnd), 56 Gy in 28 fractions to the primary anal canal tumor (PGTVp; 50 Gy in 25 fractions plus a 6 Gy/3-fraction boost), and 45 Gy in 25 fractions to the elective nodal basin (PTV). Each chemotherapy cycle comprised cisplatin 75 mg/m² (60 mg intravenously on day 1 and 50 mg on day 2) and capecitabine 1,000 mg in the morning and 1,500 mg in the evening administered on radiotherapy days in the concurrent phase and on days 1–14 during consolidation; the first two cycles were given concurrently with radiotherapy and the final two as consolidation, corresponding to a cumulative cisplatin dose of 300 mg/m². She had no history of hypertension, diabetes mellitus, thromboembolic or autoimmune disease, prior ocular disease, or hereditary optic neuropathy.

She presented with bilateral visual impairment without pain on eye movement, headache, nausea, or vomiting. At the referring hospital, ophthalmic examination revealed a best-corrected visual acuity (BCVA) of 0.6 in the right eye (OD) and 0.4 in the left eye (OS), together with bilateral optic disc edema and hyperemia. Brain magnetic resonance imaging (MRI) was recommended for further evaluation. No specific ophthalmic treatment was initiated, and the visual symptoms persisted.

Upon referral to our institution, initial ophthalmic examination showed a BCVA of 0.6 in both eyes with no improvement upon refraction, normal intraocular pressure, and a negative relative afferent pupillary defect (RAPD). Slit-lamp examination revealed no significant inflammatory exudation or aqueous flare in the anterior or posterior segments of either eye. Fundus photography demonstrated bilateral diffuse optic disc edema and hyperemia, accompanied by peripapillary radial flame-shaped hemorrhages and retinal venous dilatation and tortuosity (**Figure 1A**). At disease onset, visual field testing revealed an inferonasal defect in the right eye and a nasal defect in the left eye (**Figure 1B**). Although formal color vision testing was not performed, bedside red-green comparison showed no subjective interocular difference. Optical coherence tomography (OCT) showed diffuse thickening of the peripapillary retinal nerve fiber layer in both eyes, consistent with optic disc edema (**Figures 1C, D**). Fundus fluorescein angiography (FFA) revealed marked late-phase hyperfluorescence and leakage from both optic discs, with areas of blocked fluorescence corresponding to the retinal hemorrhages. No segmental delay or defect in retinal vascular filling was identified (**Figures 1E, F**). The angiographic findings were not typical of non-arteritic anterior ischemic optic neuropathy (NAION).

**Figure 1:**
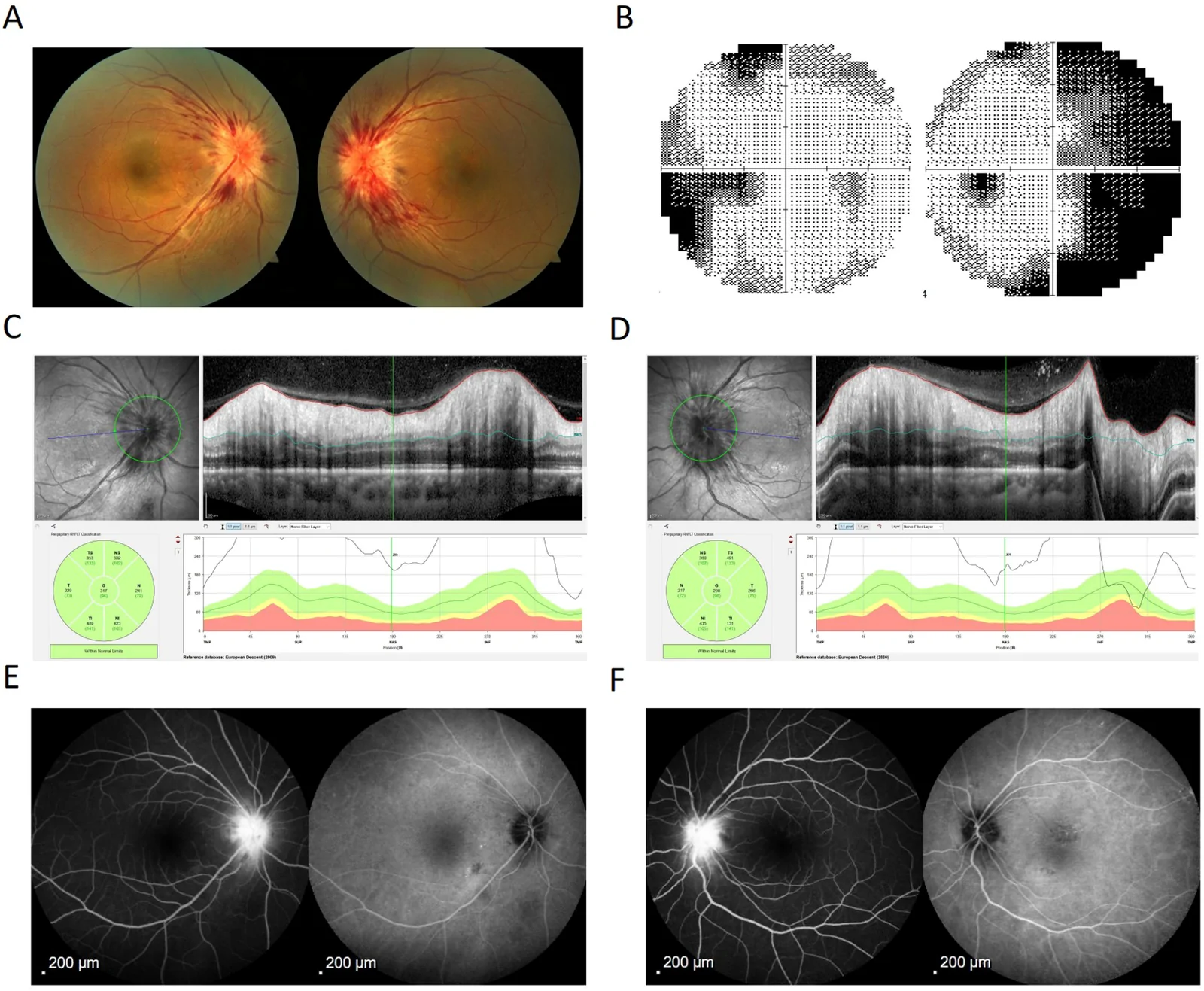
Baseline ophthalmic findings at initial presentation. **(A)** Color fundus photographs of the right eye (left image) and left eye (right image) showing bilateral diffuse optic disc edema and hyperemia, peripapillary radial flame-shaped hemorrhages, and dilated, tortuous retinal veins. **(B)** Automated visual field testing at disease onset showing an inferonasal visual field defect in the right eye (left) and a nasal visual field defect in the left eye (right). **(C, D)** Peripapillary retinal nerve fiber layer (RNFL) optical coherence tomography (OCT) of the right **(C)** and left **(D)** eye demonstrating diffuse thickening of the peripapillary RNFL consistent with optic disc edema. **(E, F)** Fundus fluorescein angiography (FFA) of the right **(E)** and left **(F)** eye in the late phases, showing marked hyperfluorescence, leakage, and staining confined to both optic discs.

To investigate potential secondary causes of optic disc edema, including toxic, infiltrative, and compressive etiologies, systemic and neuroimaging evaluations were performed. Laboratory investigations revealed macrocytic hyperchromic anemia, lymphopenia, and elevated levels of total cholesterol, low-density lipoprotein cholesterol, and γ-glutamyl transferase. Serum CA125 was mildly elevated, and anti-SOX1 antibodies were weakly positive, whereas infectious and autoimmune evaluations and the remaining tumor markers were unremarkable. Contrast-enhanced brain magnetic resonance imaging showed multiple lacunar infarcts in the bilateral frontoparietal subcortical white matter and corona radiata, as well as a pituitary lesion suggestive of a Rathke cleft cyst. No intracranial or orbital metastasis, compressive lesion, or mass effect was identified. An oncology consultation and follow-up computed tomography showed no evidence of tumor recurrence or distant metastasis. Two neurological evaluations revealed no focal neurological signs, and the intracranial imaging findings were considered unrelated to the optic disc edema. Lumbar puncture was recommended to further assess intracranial pressure but was declined by the patient. Atorvastatin 20 mg once daily was initiated for hyperlipidemia. Based on the clinical presentation and multimodal evaluation, optic neuritis, NAION, compressive or infiltrative lesions, and paraneoplastic syndrome were considered unlikely. Although intracranial hypertension could not be definitively excluded because lumbar puncture was declined, the overall findings suggested a possible association between the bilateral optic disc edema and cisplatin plus capecitabine chemotherapy.

The patient received neither glucocorticoids nor immuno-suppressants. During hospitalization, BCVA improved to 0.8 in both eyes, accompanied by marked resolution of optic disc edema and hyperemia. At the 2-month follow-up, BCVA had improved to 1.2 bilaterally. Fundus photography showed near-complete resolution of the peripapillary hemorrhages and optic disc edema, Visual field testing demonstrated improvement in the bilateral visual field defects compared with the previous examination. while OCT demonstrated reduced peripapillary RNFL thickness compared with previous scans (**Figures 2A–D**). No further progression was observed, and visual function remained well preserved without glucocorticoid or immunosuppressive therapy.

**Figure 2:**
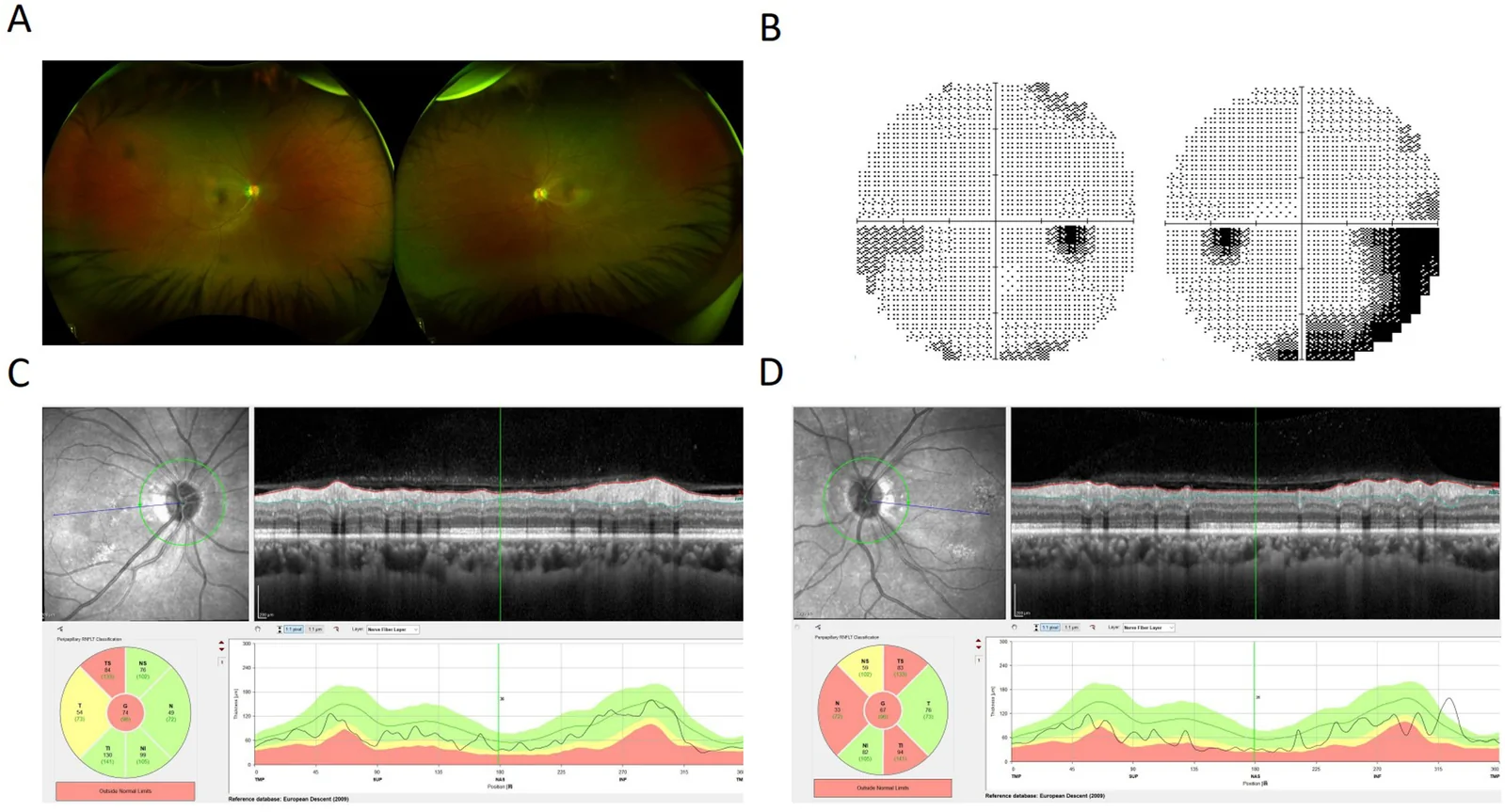
Ophthalmic findings at the 2-month follow-up. **(A)** Color fundus photographs of the right eye (left image) and left eye (right image) showing marked resolution of the bilateral optic disc edema and hyperemia, with disappearance of the peripapillary flame-shaped hemorrhages. **(B)** Follow-up 30–2 automated perimetry showed that the visual field in the right eye (left) had largely returned to normal, while the nasal visual field defect in the left eye (right) had decreased in extent compared with that at presentation. **(C, D)** Peripapillary retinal nerve fiber layer (RNFL) optical coherence tomography (OCT) of the right **(C)** and left **(D)** eye showing reduced peripapillary RNFL thickness compared with the baseline scans, consistent with resolution of the optic disc edema.

## Discussion

As cancer survival improves, uncommon ocular adverse effects of chemotherapy are receiving increasing clinical attention. Cisplatin has been associated with dose-dependent and cumulative ocular toxicity, potentially affecting the optic nerve, retina, and retinal vasculature. Capecitabine, an oral prodrug of 5-fluorouracil, has also been linked to vascular adverse effects, including vasospasm. Their concurrent administration may therefore contribute to vascular or neurotoxic injury and could potentially explain the bilateral optic disc edema observed in this patient (6). Proposed mechanisms of platinum-related ocular toxicity include ion-channel dysfunction, endothelial injury, and increased vascular permeability, with cumulative exposure potentially increasing the risk of damage (7, 15).

In this patient, ocular symptoms developed 16 days after the fourth cycle of cisplatin plus capecitabine chemotherapy, supporting a possible temporal association with cumulative drug exposure. Capecitabine-derived 5-FU has been reported to promote vascular smooth muscle contraction through protein kinase C activation and endothelin-1 upregulation, potentially causing vasospasm and impaired optic disc perfusion (16). Such vasospasm, together with cisplatin-related endothelial injury and increased vascular permeability, may have contributed to the bilateral hemorrhagic optic disc edema observed in this patient.

Fundus examination and FFA revealed bilateral optic disc edema with flame-shaped peripapillary hemorrhages and limited late-phase fluorescein leakage around the optic disc vessels, without segmental filling defects. These findings were consistent with bilateral optic disc edema with papillophlebitis-like features. A comprehensive systemic evaluation revealed no evidence of other potential causes of optic disc edema, including intracranial metastasis, raised intracranial pressure, systemic inflammatory disease, or paraneoplastic syndromes. Notably, the patient received neither glucocorticoids nor immunosuppressive therapy. During subsequent follow-up, BCVA recovered to 1.2 in both eyes, and the optic disc edema resolved spontaneously. This favorable clinical course supports a potentially reversible, chemotherapy-associated vascular or endothelial injury rather than severe irreversible ischemic damage to the optic nerve.

A review of previously published case reports indicates that the visual outcomes of platinum-associated optic disc edema are heterogeneous. Fischer et al. reported that carboplatin-associated bilateral optic disc edema resolved within weeks to months after discontinuation of the drug (17). Similarly, O’Dea et al. described four cases of oxaliplatin-related ocular neurotoxicity in which the ocular manifestations were reversible after treatment cessation (18). In contrast, Ng et al. reported persistent visual impairment in a patient with carboplatin-associated bilateral optic disc edema (6). Jayasri et al. also described a case of oxaliplatin-associated optic disc edema in which visual acuity deteriorated to 6/60, with residual visual field defects despite prompt drug withdrawal and corticosteroid treatment (19). Collectively, these reports suggest that optic disc edema associated with platinum-based chemotherapy may regress after discontinuation, whereas recovery of visual function is variable. Differences in cumulative exposure, the severity and duration of injury, the extent of optic nerve involvement, and the timing of drug withdrawal may contribute to the observed variation in prognosis.

The favorable outcome in our patient may have been related to several factors. First, the complete recovery of visual acuity and spontaneous resolution of optic disc edema suggest that the underlying injury was reversible, although its precise pathological mechanism remains uncertain. Second, the absence of further exposure to the suspected chemotherapeutic agents may have limited additional ocular injury. Third, the absence of vascular risk factors, including hypertension and diabetes mellitus, may have contributed to the favorable clinical course.

Evaluation of new optic disc edema after chemotherapy should be individualized and multidisciplinary. Contrast-enhanced cranial MRI can help identify mass lesions, infiltration, and alternative neurological causes. Measurement of cerebrospinal fluid opening pressure is needed when raised intracranial pressure remains clinically plausible. FFA, OCT, visual fields, and color vision can help characterize the phenotype and monitor its course. Paraneoplastic antibodies should be interpreted in their clinical context, particularly when a result is weakly positive and isolated.

Patients receiving potentially ocular-toxic chemotherapy should be advised to report new visual symptoms promptly. This single case does not justify routine baseline OCT or fundus imaging for all platinum-treated patients, nor does it support automatic cessation of essential therapy. Baseline and follow-up imaging may be considered for selected high-risk patients or regimens. Cross-sectional OCT/OCTA findings suggest a possible association between cumulative cisplatin exposure and retinal or RNFL thinning (14). OCT, with OCTA when available, may be considered during follow-up, particularly for patients receiving higher cumulative doses or reporting visual symptoms. Decisions to withhold, modify, or resume chemotherapy should balance the ocular findings, oncologic benefit, alternative treatments, and patient preferences.

This report has several limitations. First, intracranial hypertension could not be definitively excluded because lumbar puncture and cerebrospinal fluid opening pressure measurement were not performed. Second, color vision was assessed only by subjective bedside red–green comparison. Third, as this was a single case without rechallenge, drug-level measurements, or a clear dose–response relationship, causality could not be established, nor could the responsible agent be identified.

## Conclusion

Bilateral optic disc edema may represent a rare and potentially reversible ocular adverse event associated with cisplatin plus capecitabine therapy. New visual symptoms after chemotherapy warrant prompt ophthalmic and systemic evaluation. Treatment decisions should be individualized through multidisciplinary assessment.

## Data Availability

The raw data supporting the conclusions of this article will be made available by the authors, without undue reservation.
